# Isolation, identification, and evaluation of the antioxidant properties of lactic acid bacteria strains isolated from meat environment

**DOI:** 10.1371/journal.pone.0327225

**Published:** 2025-07-01

**Authors:** Anna Łepecka, Piotr Szymański, Anna Okoń

**Affiliations:** Department of Meat and Fat Technology, Prof. Waclaw Dabrowski Institute of Agricultural and Food Biotechnology—State Research Institute, Warsaw, Poland; University of Johannesburg, SOUTH AFRICA

## Abstract

Oxidative stress is a condition in which the body loses balance between the production of free radicals and the body’s ability to neutralize them. The role of antioxidants is to protect cells and tissues from the harmful effects of excessive amounts of free oxygen radicals. Lactic acid bacteria (LAB) can exhibit significant antioxidant properties which is the subject of research by many scientists. The aim of the work was isolation, phenotypic and genotypic identification, and evaluation of the antioxidant activity of twenty-one bacterial strains from raw fermented beef hams and the environment of a meat factory. The bacteria were screened *in vitro* by investigating their DPPH (1,1-diphenyl-2-picrylhydrazyl) and ABTS (2,2′-azino-bis(3-ethylbenzothiazoline-6-sulfonic acid)) free radical scavenging activity, superoxide anion tests, hydroxyl radical resistance, superoxide dismutase and catalase activity, and hydrogen peroxide resistance. As a result of the conducted research, 21 bacterial strains were isolated. They were assigned to *Lactiplantibacillus plantarum* (14), *Lactiplantibacillus pentosus* (3), *Lactiplantibacillus argentoratensis* (2), *Lacticaseibacillus paracasei* (1), and *Pediococcus pentosaceus* (1). The strains were compared with each other and some of them were able to scavenge free radicals DPPH (1.08–36.91%), ABTS (11.24–51.05%), and superoxide anions (3.04–96.70%). Furthermore, resistance to high concentrations of hydrogen peroxide (0.4–1.0 mM H_2_O_2_) and hydroxyl radicals (25.90–99.22%) has been demonstrated. Some strains produced superoxide dismutase, while none of them produced catalase. The findings indicated that some LAB strains could be promising starter candidates with antioxidant properties.

## Introduction

Oxidative stress is a state of imbalance between the intensity of oxidative processes that stimulate the formation of free oxygen radicals and the antioxidant capacity of the body to remove them [1]. An excess of oxidants can lead to cell and DNA damage, as well as protein aggregation, cell membrane dysfunction, lipid peroxidation, carbonyl adduct formation, nitration, and sulfoxidation [2,3]. These pathological changes disrupt physiological redox signaling, which in turn leads to defective hydrogen peroxide signaling in cellular processes [2]. In physiological processes, free radicals are under the strict control of the body, as a result of the action of enzymatic and non-enzymatic defense mechanisms. High oxygen levels can lead to the formation of reactive oxygen species (ROS), as well as reactive nitrogen species (RNS), reactive chlorine species (RCS), and reactive sulfur species (RSS) [1,4,5].

Free radicals play a dual role in the body, which are both positive and negative. In low concentrations, they participate in normal physiological processes. They are essential in metabolic processes, participating in the immune response, where they inhibit the growth of pathogens, participating in the transmission of signals between cells, and in the regulation of inflammatory processes [6]. On the other hand, their accumulation leads to oxidative/nitrosative stress. As a result, damage occurs to nucleic acids, proteins, and membrane lipids, causing cell death or loss of function, and can also lead to mutagenesis [7]. The most reactive forms are considered to be: hydroxyl radical (OH^•^), perhydroxyl radical (HO_2_^•^), superoxide anion radical (O_2_^•¯^), hydrogen peroxide (H_2_O_2_), singlet oxygen (^1^O_2_), hypochlorous acid (HOCl), nitric oxide radical (NO^•^), hypochlorite radical (OCl^•^), peroxynitrite (ONOO^-^), and peroxyl radicals (ROO^•^) [4,5]. The accumulation of free radicals leads to the development of chronic diseases and aging such as cardiovascular diseases [8], diabetes [9], psychiatric diseases (depression, schizophrenia) [10,11], chronic kidney disease [12], lung disease [13], neurodegenerative disorders (Alzheimer’s disease, Parkinson’s disease, amyotrophic lateral sclerosis, Down syndrome) [5,14], metabolic syndrome [15], or cancer [3].

Antioxidants are chemical compounds that neutralize free radicals and other molecules that can damage cells, proteins, lipids, and DNA. Their task is to prevent, inhibit, and reduce oxidation processes [16]. The concentration of reactive molecules in the cellular system can be reduced by antioxidants. Strategies for managing oxidative status may include several aspects. First of all, it is the use of natural antioxidants, such as vitamin C, vitamin E, polyphenols, or flavonoids. Lifestyle changes, including a healthy diet, regular physical activity, and avoiding stress, are also important. There are also pharmacological drugs aimed at improving mitochondrial function or neutralizing ROS. The need for a better understanding of the action of antioxidants in the context of chronic diseases has also been noted [8,17,18].

Lactic acid bacteria (LAB) are commonly used in the production of fermented foods of both plant origin (fermented vegetables, fruit beverages, soy, legumes, and cereal products) and animal origin (dairy products, fermented meat, and fish). To maintain the optimal functioning of the body, the consumption of natural antioxidants is becoming an increasingly popular option that allows for improving the mechanisms of protection against free radicals [4,19]. However, to ensure the appropriate quality of products, and therefore the appropriate number of bacteria, as well as the health-promoting effect they can cause, it is necessary to conduct strain screening tests. The aim is to isolate bacteria with the best functional and technological properties. The use of LAB resistant to stress conditions is of great importance for maintaining the appropriate viability of these bacteria when they colonize the gastrointestinal tract. LAB are anaerobic and therefore do not synthesize an active electron transport chain, which is crucial when they are in aerobic conditions [1].

The role of lactic acid bacteria as natural antioxidants is a new and rapidly developing area of research. They act through a number of mechanisms that allow them to neutralize free radicals, protect cells from oxidative stress, and support the host’s antioxidant defense system [1]. Some LAB strains can directly scavenge ROS, such as superoxide anion radical, hydrogen peroxide, or hydroxyl radicals. This mechanism involves binding free radicals by compounds present on the bacterial cell surface or secreted metabolites. Another mechanism involves the synthesis of enzymes that catalyze the decomposition of ROS, such as superoxide dismutase, catalase, or glutathione peroxidase. Not all LAB produce these enzymes, but many strains have been selected for their enzymatic activity. Another mechanism involves the chelation of transition metal ions (iron, copper). Binding these metals and inhibiting the Fenton reaction allows for reducing environmental toxicity. In addition, LAB can affect the expression of genes responsible for antioxidant defense, as well as synthesize antioxidant compounds. Additionally, during food fermentation, LAB can transform ingredients into bioactive compounds with even greater antioxidant potential [1,20]. The antioxidant activity of LAB strains has been tested in numerous studies [21–25]. Our previous study [26] proved that fermented meat products are an excellent source of LAB strains with promising antioxidant properties. We also tested some of them in fermented sausages [27]. Moreover, antioxidants, thanks to their ability to reduce oxidative stress, play an important role in maintaining human health, preventing and treating diseases [28–31]. Understanding the mechanisms of free radical reactions occurring *in vitro* in model biological systems can contribute to understanding the processes and can also enable counteracting the negative effects of oxidative stress. The study aimed to isolate, identify, and assess the antioxidant activity of lactic acid bacteria strains isolated from meat environment. The scope of this study is not only to confirm the ability of the strains to neutralize free radicals but also to bring a new perspective to previous studies by analyzing their action both in the context of functional food and possible therapeutic application. The unusual nature of this work consists in departing from the classical perception of lactic acid bacteria only as fermentative or probiotic microorganisms, but also in presenting them as possible bioactive components with antioxidant and health-promoting effects.

## Materials and methods

### Isolation

The material for the isolation of lactic acid bacteria strains was raw fermented beef hams and the meat factory production environment. The meat processing plant is located in Poland, in the Subcarpathian Voivodeship. It produces organic meat products, without additives and starter cultures.

The methodology for the isolation of microorganisms was taken from the research by Zielińska et al. [32]. In the case of beef hams (5 products from different batches), 10 g of meat product was weighed into a bag with a side filter (BagFilter^®^ 400P, Interscience, France) and 90 mL of buffered peptone water (Biomaxima, Lublin, Poland) was added, and then, homogenized in a paddle homogenizer (Stomacher Lab-Blender 400, Gemini BV, Netherlands) for 5 min. Subsequently, 0.1 mL of homogenate was collected and inoculated onto Petri dishes with MRS agar (de Man, Rogosa, Sharpe agar, Biomaxima). The plates were incubated for 72 h at 30 °C. Then, single, different colonies were randomly isolated and transferred to MRS broth (de Man, Rogosa, Sharpe broth, Biomaxima). The purity of the cultures was checked each time by inoculating them on fresh MRS agar. In the case of the meat factory production environment, the swab method was used. A sterile swab soaked in 10 mL buffered peptone water was used to make a 5x5 cm swab from the production surface (production trolley, meat cutting table, production table, meat grinder). The swab was transferred to a sterile package with MRS broth and immediately transported to the laboratory, where the MRS broth was incubated for 24 h at 37 °C. Then 0.1 mL of broth was taken and inoculated onto Petri dishes with MRS agar. The next steps were performed as in the case of isolates from beef hams.

### Phenotypic characterization

The Gram staining of bacteria was prepared based on the publication of Paray et al. [33]. A bacterial colony was placed on a glass slide with a drop of distilled water and then flame-fixed. The slide was covered with crystal violet and left for 1 min. Then it was rinsed with distilled water, covered with Lugol’s iodine, and left for another 1 min. Then it was rinsed with distilled water and covered with 95% ethyl alcohol for 10 s. Immediately rinsed with distilled water and covered with safranin for 30–60 s. Then the solution was rinsed with distilled water and dried with filter paper. The finished slide was observed under a microscope (Nikon ECLIPSE E200, Nikon Instruments Inc., Melville, USA) at 1000x magnification with the addition of immersion oil. Gram-positive bacteria stain purple, while Gram-negative bacteria stain red. Chemical reagents for Gram staining were purchased from Pol-Aura (Morąg, Poland). The catalase test was performed with the addition of hydrogen peroxide, each time spotting a 3% hydrogen peroxide solution (Chempur, Piekary Śląskie, Poland) on single colonies of microorganisms located on MRS agar plates [32]. The gas bubbles that are released indicate the presence of catalase (catalase-positive bacteria). The absence of gas bubbles defines the bacteria as catalase-negative. Biochemical tests API^®^ 50 CH (bioMérieux, Marcy-l’Étoile, France) were performed according to the manufacturer’s instructions in combination with API^®^ 50 CHL Medium (bioMérieux). These tests allow for preliminary assignment of bacteria to genus and species based on carbohydrate metabolism.

### Genotypic characterization

The methodology is derived from the internal procedure of the A&A Biotechnology laboratory. Genomic DNA was isolated using the Genomic Mini AX Bacteria+ kit (A&A Biotechnology, Gdansk, Poland) with an additional mechanical lysis of the sample in a FastPrep device using zirconium beads. A polymerase chain reaction (PCR) was performed. The composition of the reaction mixture was as follows: PCR Mix Plus HGC (A&A Biotechnology), Starter For (GAG TTT GAT CCT GGC TCA G) at a concentration of 100 μM, Starter Rev (ACG GCT ACC TTA CGA CTT) at a concentration of 100 μM, genomic DNA (5 μL), water to 50 μL. The temperature-time profile was as follows: initial denaturation (94 °C, 120 s, number of cycles 1), denaturation (94 °C, 30 s, number of cycles 30), primer annealing (58 °C, 30 s, number of cycles 30), extension (72 °C, 90 s, number of cycles 30), final extension (72 °C, 300 s, number of cycles 1). DNA fragments obtained as a result of the amplification reaction were purified using the Clean-Up AX kit (A&A Biotechnology). The PCR products were suspended in a 10 mM Tris-HCl buffer (A&A Biotechnology), diluted to 50 ng/μL, and sent for sequencing. Gene nucleotide sequences were deposited in the GenBank database.

### Phylogenetic tree

The phylogenetic tree was constructed using MEGA11 (Molecular Evolutionary Genetics Analysis Software version 11.0.13). Neighbor-joining was used as a statistical method. The bootstrap method was used as a test of the phylogeny. No. of bootstrap replications was 1000 [34].

### Antioxidant activity assay

#### Bacteria cultures.

Twenty-one strains of food-origin bacteria were used in this study. The isolates originate from Prof. Waclaw Dabrowski Institute of Agricultural and Food Biotechnology – State Research Institute’s Collection of Industrial Microorganisms. Antioxidant properties were compared with the reference probiotic strain *Lacticaseibacillus (L.) rhamnosus* GG (positive control; GenBank accession AP011548) and the non-antioxidant strain *Escherichia (E.) coli* DH5α (negative control; GenBank accession CP017100) [35]. *L. rhamnosus* GG was purchased from Argenta (Poznań, Poland), while *E. coli* DH5α was purchased from Carolina BioSystems (Prague, Czech Republic).

Lactic acid bacteria (LAB) isolates suspended in MRS broth and *E. coli* isolate suspended in LB broth (Luria-Bertani broth, Biomaxima) were stored with the addition of glycerol (20%, v/v; Chempur) at −80 °C. Cell-Free Supernatant (CFS) or Intact cells (IC) were used to study.

#### Cell-free supernatant (CFS).

CFS was prepared according to the methodology of Shi et al. [35]. The LAB cultures were grown in fresh MRS broth, while the *E. coli* cultures were grown in LB broth at 37 °C for 18 h. Then, the culture was centrifuged in a centrifuge (MPW-56, MPW Med. Instruments, Warsaw, Poland) at 6000 *x* g for 10 min and sterilized using a MF-Millipore™ Membrane Filter (0.22 µm, 13 mm; Merck, Darmstadt, Germany).

#### Scavenging of DPPH-free radical.

The CFS obtained from each bacterial strain was used for the studies. A fresh 0.2 mM solution of DPPH (2,2-diphenyl-1-picrylhydrazyl; Merck) in methanol (99.8%; Chempur) was prepared [35]. Then, a mixture of 1 mL of DPPH and 0.8 mL of CFS was made. The mixture was left in darkness for 30 min and then, the absorbance was measured spectrophotometrically at 517 nm (Hitachi U‐2900, Tokyo, Japan). In the blank solution, CFS was replaced by pure MRS broth (or LB broth). The activity was calculated according to the equation:


DPPH (%) = (A0 – A1 / A0) × 100% (1)

A_1_ – absorbance of the mixture with CFS; A_0_ – absorbance of the mixture where CFS was replaced by MRS broth (or LB broth)

#### ABTS radical scavenging activity.

The CFS obtained from each bacterial strain was used for the studies. ABTS ((2,2′-azino-bis(3-ethylbenzothiazoline-6-sulfonic acid); Sigma Aldrich, Poznań, Poland) radical scavenging activity was studied using the method described by Shi et al. [35]. Briefly, a stock solution of ABTS^+^ radical cation (7 mM ABTS with 2.45 mM potassium persulfate; Sigma Aldrich) was prepared and the mixture was kept for 16 h in darkness at room temperature. The ABTS^+^ solution was diluted with methanol to an absorbance of 0.700 ± 0.020 at 734 nm. Then, 100 µL of CFS was mixed with 3.0 mL of ABTS^+^ solution and the absorbance was measured at 734 nm (Hitachi U‐2900). In the blank solution, CFS was replaced by pure MRS broth (or LB broth). The results were calculated according to the equation:


ABTS+ (%) = (1 – A1 / A0) × 100% (2)

where: A_1_ – absorbance of the mixture with CFS; A_0_ – absorbance of the mixture, where CFS was replaced by MRS broth (or LB broth)

#### Superoxide anion scavenging test.

The CFS obtained from each bacterial strain was used for the studies. The superoxide anion scavenging test was performed using the method described by Gao et al. [36] with minor modifications from Shi et al. [35]. A reaction mixture containing 0.8 mL of CFS and 0.2 mL of Tris–HCl buffer (0.1 M, pH 8.0; Sigma Aldrich) was prepared. The absorbance was measured at 320 nm. Then, 0.1 mL of pyrogallol solution (3 mM; Sigma Aldrich) was added and the absorbance was measured again at 320 nm. Anion resistance was determined in % according to the equation:


superoxide anion resistance (%) = (1−(A1−A2)/A0) × 100% (3)

where: A_1_ – absorbance of CFS containing pyrogallol; A_2_ – absorbance of CFS without pyrogallol; A_0_ – absorbance of pure MRS broth (or LB broth) containing pyrogallol

#### Hydroxyl radical resistance.

The study of hydroxyl radical resistance was performed based on Shi et al. [35]. The CFS obtained from each bacterial strain was used for the studies. Hydroxyl radical generation was carried out in a solution containing 1 mL of 1.10-phenanthroline (0.75 mM; Sigma Aldrich), 1.5 mL of sodium phosphate buffer (pH 7.4; 0.15 M; Sigma Aldrich), 1 mL of FeSO_4_ (0.75 mM; Sigma Aldrich), 1 mL of H_2_O_2_ (0.01%, v/v; Sigma Aldrich) and 1.0 mL of CFS. Then, the mixture was incubated at 37 °C for 30 min and the absorbance was measured spectrophotometrically at 536 nm (Hitachi U‐2900). The change in absorbance of the reaction mixture indicates the ability of the strains to scavenge hydroxyl radicals. The results were expressed as:


resistance to hydroxyl radicals (%) = (A1 – A0)/(A2 − A0) × 100% (4)

where: A_1_ – absorbance of the mixture with CFS, A_2_ – absorbance of the mixture, where CFS and H_2_O_2_ were replaced by MRS broth (or LB broth), A_0_ – absorbance of the mixture, where CFS was replaced by MRS broth (or LB broth)

#### Intact cells (IC).

Previously revived isolates incubated in MRS broth (or LB broth) were transferred to fresh MRS broth or LB broth and incubated at 37 °C for 24 h to provide intact cells (IC). IC without centrifugation and filtration were used for subsequent studies [35].

#### Superoxide dismutase (SOD) activity.

The methodology was adapted from Tomusiak-Plebanek et al. [37]. SOD activity expressed in U/mL was measured using DetectX® Superoxide Dismutase (SOD) Colorimetric Activity Kit (Arbor Assays, Michigan, USA). Assays were performed according to the manufacturer’s instructions.

A semi-quantitative method using test strips was used to assess the ability of the strains to degrade superoxide anion radicals. Hydrogen peroxide was measured using the Peroxide Test MQuant^®^ (Merck) with an H_2_O_2_ detection scale from 0 to 100 mg/L (0-1-3-10-30-100 mg/L). The tested LAB and *E coli* cultures were suspended in MRS broth (or LB broth) and incubated at 37 °C for 24 h in aerobic conditions. Hydrogen peroxide concentration was measured at time 0 and after 24 h of incubation [37].

#### Catalase (CAT) activity.

The methodology was adapted from Tomusiak-Plebanek et al. [37] with minor modifications. Hydrogen peroxide was measured using the analytical test strips Peroxide Test MQuant^®^ (Merck) with an H_2_O_2_ detection scale from 0 to 100 mg/L (0-1-3-10-30-100 mg/L). LAB and *E. coli* cultures were prepared in 1 mL of MRS broth (or LB broth) at 37 °C for 24 h under aerobic conditions. The supernatant was then centrifuged at 3000 x g for 15 min at 4 °C. The supernatant was decanted and the remaining cell pellet was suspended in 1 mL of fresh MRS broth (or LB broth) with hydrogen peroxide (30 mg/L; Merck) and incubated at 37 °C for 24 h under aerobic conditions. The hydrogen peroxide concentration was measured at time 0 and after 24 h of incubation.

#### Hydrogen peroxide resistance.

Resistance was checked according to the methodology of Li et al. [23]. Strains were incubated in MRS broth (or LB broth) at 37 °C for 16 h. Then, 1% (v/v) of the broth was transferred to fresh MRS broth (or LB broth) supplemented with 0.0, 0.4, 0.7, or 1.0 mM hydrogen peroxide (Sigma Aldrich) and re-incubated at 37 °C for 8 h. Cell growth was measured spectrophotometrically (Hitachi U-2900) at 600 nm. The results were given as optical density (OD_600_).

### Statistical analysis

The study was performed in three independent replicates. The obtained results were presented as mean and standard deviation. To analyze the effects, a one-way analysis of variance (ANOVA) was performed, with a significance level of *p* < 0.05. Tukey’s HSD post-hoc test was used to compare pairs of means. Hydrogen peroxide resistance was analyzed using conditional formatting (Excel 2016, Microsoft Corporation, USA). A clustering model was realized through Ward’s Clustering Method with Euclidean squared distance. Statistica 13.3 (TIBCO Software Inc., Palo Alto, USA) was used for the calculations.

## Results and discussion

### Isolation and identification

As a result of the conducted research, twenty-one bacterial isolates were collected. Table 1 presents the results of the phenotypic identification of the strains. All isolates were Gram-positive, and catalase-negative, 20 rods of different sizes and 1 small cocci were observed. API^®^ 50 CH tests distinguished 4 species: *Lactiplantibacillus (L.) plantarum* (17 isolates), *Lactiplantibacillus (L.) pentosus* (1 isolate), *Pediococcus (Pd.) pentosaceus* (1 isolate) and *Lacticaseibacillus (L.) paracasei* (2 isolates). It was found that the tested LAB strains metabolize typical carbohydrates for the genera *Lactobacillus* and *Pediococcus*.

**Table 1:** Characteristics of the tested isolates.

No	Isolate symbol	Isolation source	Gram staining, morphology	Catalase activity	API^®^ 50 CH test
1	S1A	Beef ham (I)	Gram-positive, non-motile, non-spore-forming, short, thin rods, found singly, in pairs, or in chains, length 4–5 μm, width 1 μm	catalase-negative	*L. plantarum*
2	S1B	Beef ham (I)	Gram-positive, non-motile, non-spore-forming, short, thick rods, found singly, in pairs, or in chains, length 4–5 μm, width 1.5 μm	catalase-negative	*L. pentosus*
3	S1C	Beef ham (I)	Gram-positive, non-motile, non-spore-forming, short, thick rods, found singly, in pairs, or in chains, length 3–5 μm, width 1.5 μm	catalase-negative	*L. plantarum*
4	S2A	Beef ham (II)	Gram-positive, non-motile, non-spore-forming, short, thick rods, found singly, in pairs, or in chains, length 3–4 μm, width 1.5 μm	catalase-negative	*L. plantarum*
5	S2B	Beef ham (II)	Gram-positive, non-motile, non-spore-forming, short, thick rods, found singly, in pairs, or in chains, length 3–4 μm, width 1.5 μm	catalase-negative	*L. plantarum*
6	S3A	Beef ham (III)	Gram-positive, non-motile, non-spore-forming, short, thick rods, found singly, in pairs, or in chains, length 2–3 μm, width 2 μm	catalase-negative	*L. plantarum*
7	S3B	Beef ham (III)	Gram-positive, non-motile, non-spore-forming, short, thick rods, found singly, in pairs, or in chains, length 2–3 μm, width 2 μm	catalase-negative	*L. plantarum*
8	S4A	Beef ham (IV)	Gram-positive, non-motile, non-spore-forming, short, thick rods, found singly, in pairs, or in chains, length 2–3 μm, width 1.5 μm	catalase-negative	*L. plantarum*
9	S4B	Beef ham (IV)	Gram-positive, non-motile, non-spore-forming, short, thick rods, found singly, in pairs, or in chains, length 2–3 μm, width 2 μm	catalase-negative	*L. plantarum*
10	S5A	Beef ham (V)	Gram-positive, non-motile, non-spore-forming, short, thick rods, found singly, in pairs, or in chains, length 2–3 μm, width 2 μm	catalase-negative	*L. plantarum*
11	S5B	Beef ham (V)	Gram-positive, non-motile, non-spore-forming, long, thick rods, found singly, in pairs, or in chains, length 2–4 μm, width 1.5 μm	catalase-negative	*L. plantarum*
12	OP1	Production trolley	Gram-positive, non-motile, non-spore-forming, short, thick rods, found singly, in pairs, or in chains, length 2–5 μm, width 1.5 μm	catalase-negative	*L. paracasei*
13	OP2	Meat cutting table	Gram-positive, non-motile, non-spore-forming, small cocci, forms tetrads, 0.5–1 μm diameter	catalase-negative	*Pd. pentosaceus*
14	OP3	Meat cutting table	Gram-positive, non-motile, non-spore-forming, long, thick rods, found singly, in pairs, or in chains, length 3–6 μm, width 2 μm	catalase-negative	*L. paracasei*
15	OP4	Production table	Gram-positive, non-motile, non-spore-forming, short, thick rods, found singly, in pairs, or in chains, length 2–4 μm, width 1.5 μm	catalase-negative	*L. plantarum*
16	OP5	Meat cutting table	Gram-positive, non-motile, non-spore-forming, short, thick rods, found singly, in pairs, or in chains, length 2–4 μm, width 1.5 μm	catalase-negative	*L. plantarum*
17	OP6	Meat grinder	Gram-positive, non-motile, non-spore-forming, long, thick rods, found singly, in pairs, or in chains, length 4–6 μm, width 1.5 μm	catalase-negative	*L. plantarum*
18	OP7	Meat grinder	Gram-positive, non-motile, non-spore-forming, long, thick rods, found singly, in pairs, or in chains, length 4–6 μm, width 1.5 μm	catalase-negative	*L. plantarum*
19	OP8	Meat grinder	Gram-positive, non-motile, non-spore-forming, long, thick rods, found singly, in pairs, or in chains, length 4–6 μm, width 1.5 μm	catalase-negative	*L. plantarum*
20	OP9	Production table	Gram-positive, non-motile, non-spore-forming, short, thick rods, found singly, in pairs, or in chains, length 2–3 μm, width 1.5 μm	catalase-negative	*L. plantarum*
21	OP10	Production table	Gram-positive, non-motile, non-spore-forming, short, thick rods, found singly, in pairs, or in chains, length 2–4 μm, width 1.5 μm	catalase-negative	*L. plantarum*

I, II, III, IV, V – production batches of beef hams.

Genotypic identification (Table 2) allowed the assignment of strains to species: *Lactiplantibacillus (L.) plantarum* (14 isolates), *Lactiplantibacillus (L.) pentosus* (3 isolates), *Lactiplantibacillus (L.) argentoratensis* (2 isolates), *Pediococcus (Pd.) pentosaceus* (1 isolate), and *Lacticaseibacillus (L.) paracasei* (1 isolate). The similarity to the reference species in BLAST was 99.03–100%. All strain sequences were deposited in the GenBank database. All identified strains are deposited in Prof. Wacław Dąbrowski Institute of Agricultural and Food Biotechnology – State Research Institute’s Collection of Industrial Microorganisms. Strain *L. plantarum* OP5 is in patent deposit (Patent application no P. 449994 – Patent Office of the Republic of Poland).

**Table 2:** Genetic identification of bacterial strains.

Strain symbol	Genus and species	BLAST similarity (%)	GenBank accession
S1A	*Lactiplantibacillus plantarum*	100.00	OP782669
S1B	*Lactiplantibacillus plantarum*	99.63	OP783985
S1C	*Lactiplantibacillus plantarum*	99.87	OP784257
S2A	*Lactiplantibacillus plantarum*	100.00	OP784365
S2B	*Lactiplantibacillus pentosus*	99.85	OP784389
S3A	*Lactiplantibacillus plantarum*	99.87	OP793630
S3B	*Lactiplantibacillus pentosus*	99.61	OP793642
S4A	*Lactiplantibacillus plantarum*	99.52	OP793679
S4B	*Lactiplantibacillus pentosus*	99.40	OP793706
S5A	*Lactiplantibacillus plantarum*	100.00	OP793803
S5B	*Lactiplantibacillus argentoratensis*	99.88	OP793843
OP1	*Lactiplantibacillus plantarum*	100.00	OP793883
OP2	*Pediococcus pentosaceus*	100.00	OP793890
OP3	*Lacticaseibacillus paracasei*	100.00	OP793889
OP4	*Lactiplantibacillus plantarum*	99.03	OP793897
OP5*	*Lactiplantibacillus plantarum*	99.76	OP793894
OP6	*Lactiplantibacillus plantarum*	100.00	OP800913
OP7	*Lactiplantibacillus argentoratensis*	100.00	OP800914
OP8	*Lactiplantibacillus plantarum*	100.00	OP804244
OP9	*Lactiplantibacillus plantarum*	99.86	OP805595
OP10	*Lactiplantibacillus plantarum*	100.00	OP805599

*Patent deposit.

The microbiota of fermented meat products varies depending on processing technology, production site, and product type. The dominant microorganisms in meat fermentation are invariably lactic acid bacteria (LAB), coagulase-negative staphylococci (CNS), yeasts, molds [38,39], and filamentous fungi [40]. In the study by Rzepkowska et al. [41], bacterial strains were isolated from raw-ripened meat products such as sausage, pork roast, and gammon. The following species were identified: *L. plantarum*, *Levilactobacillus (L.) brevis,* and *Pd. pentosaceus*. In the study of Aquilanti et al. [42] LAB were isolated from meat skewer, minced pork meat, fresh pork sausage, and poultry carcasses. The species were identified: *Lactococcus (Lc.) garvieae*, *Lc. lactis* subsp. *lactis*, *Lactobacillus* (now *Lactiplantibacillus*) *plantarum*, *Lactobacillus (L.) johnsonii*, *Ligilactobacillus (L.) salivarius*, *Limosilactobacillus (L.) reuteri*, *L. brevis*, *Lactobacillus (L.) crispatus*. According to da Costa et al. [43], LAB strains isolated from meat and meat products are of lesser interest to scientists than those originating from milk and dairy products. Particular attention is paid to the bacteriocinogenic properties of these bacteria [44]. However, fermented meat products are an excellent source of new LAB strains with unique properties, not only antimicrobial but also antioxidant, as proven in this manuscript.

**Fig 1:**
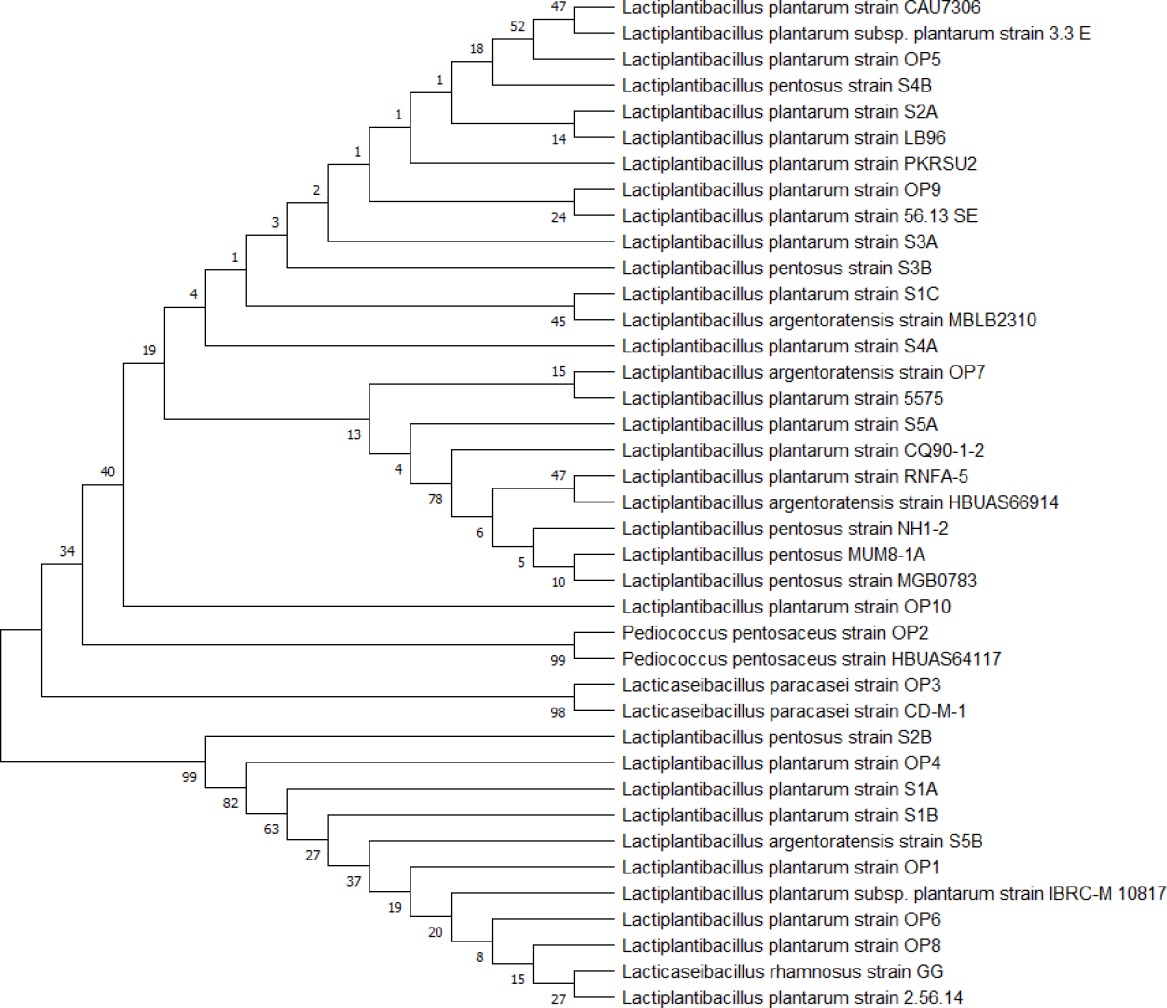
Phylogenetic tree of the studied strains. The phylogenetic tree of lactic acid bacteria strains shows the evolutionary relationships between different species of these bacteria. Relationships were determined based on the 16S rRNA gene sequence.

To determine the phylogenetic position and relatedness of the studied strains in comparison to other strains, we constructed a neighbor-joining phylogenetic tree consisting of 1000 bootstrap replicates. The phylogenetic analysis is shown in Fig 1. The phylogenetic tree shows the relationships between sequences or species of microorganisms. The analysis used sequences of twenty-one tested LAB strains, *L. rhamnosus* GG (probiotic), and strains to which similarity was demonstrated during sequencing and assignment of isolates to genera and species. Bacterial strains of the following species have been proposed: *Lactiplantibacillus plantarum*, *Lactiplantibacillus pentosus*, *Lactiplantibacillus argentoratensis*, *Pediococcus pentosaceus*, *Lacticaseibacillus paracasei*, and *Lacticaseibacillus rhamnosus*.

### Antioxidant activity

There are currently no uniform standards for testing the antioxidant properties of bacterial strains. However, methods have been developed to assess these properties, consisting of the detection of free radicals and metal ions, enzyme activity tests, or tests for detecting end products [1]. Therefore, as indicated by Garcia-Gonzalez et al. [45], it is recommended to assess antioxidant properties with more than one test. According to Gulcin & Alwasel [46], at least three different methods should be used. The study of antioxidant activity is a very complex process, therefore it is advisable to choose different research methods, even those that are not strictly correlated with each other. In this way, it is possible to better understand the mechanisms of action of antioxidants [47]. In this study, we proposed several methods to investigate antioxidant activity.

### Scavenging of DPPH-Free Radical

**Fig 2:**
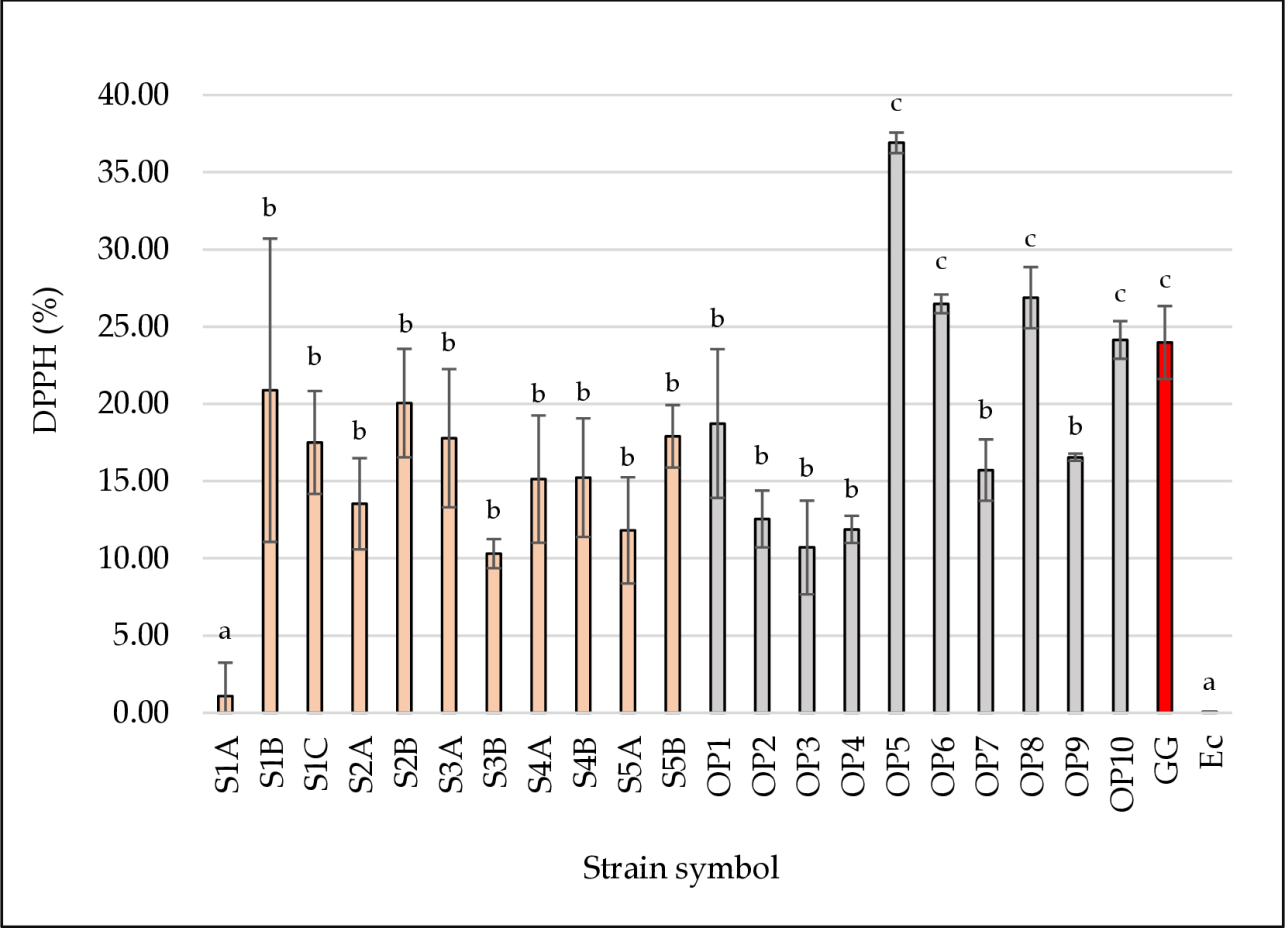
Antioxidant activity by the DPPH method of the tested LAB strains. The figure presents the ability of bacterial strains to capture free DPPH radicals. Strains isolated from beef hams are marked in pink. Strains isolated from the meat factory environment are marked in grey. The reference probiotic strain *L. rhamnosus* GG (positive control) is marked in red. The reference strain *E. coli* DH5α (negative control) is marked in green. ^a,b,c^ Means in the same column followed by different superscripts are significantly different (*p* < 0.05).

The DPPH method is the most probable, popular, and widely used method to determine antioxidant activity [46]. The method is based on the spectrophotometric measurement of the ability of antioxidants to scavenge DPPH free radicals [48]. Fig 2 shows the antioxidant activity of LAB strains determined by the DPPH method. It was found that the strains *L. plantarum* OP5, OP6, OP8, and OP10 had significantly the highest antioxidant activity (24.14–36.91%; *p* < 0.05). These four strains isolated from the production environment showed higher antioxidant activity by the DPPH method than the reference strain *L. rhamnosus* GG (23.98%). It is assumed that DPPH above 30% indicates high antioxidant activity [49]. The mechanism of the action of lactobacilli is their ability as antioxidants to scavenge DPPH radicals, which is attributed to their ability to donate hydrogen [50]. In the study by Mu et al. [51] thirteen strains of lactobacilli were tested for DPPH scavenging activity. As in our study, *L. rhamnosus* GG was used as a positive control. Moreover, a similar antioxidant activity of over 30% was obtained. Six strains of the species *Lactobacillus casei* (now *Lacticaseibacillus casei*) and *L. plantarum* showed higher or similar antioxidant activity compared to *L. rhamnosus* GG. In the study by Shi et al. [35], twenty-three strains from different LAB species were tested for DPPH free radical scavenging. The obtained results were in the range of 2.79–39.30%. The authors concluded that substances located on the cell surface significantly affect the ability to actively scavenge free radicals. Similar results of the DPPH radical-scavenging capacity of LAB isolates were obtained in studies by Hernández-Delgado et al. [52]. The percentage of DPPH inhibition was 27.12–43.99, and statistically significantly the highest activity was shown by *L. plantarum* LM17 (*p* < 0.05). Agave isolates were compared with the commercial strain *L. plantarum* Lp115 (37.39%). The *in vitro* DPPH radical scavenging activity of strains derived from traditional Chinese fermented foods was investigated [23]. The antioxidant activity was shown to be dose-dependent. The higher the number of LAB, the higher the ability to capture free radicals. In addition, the *L. plantarum* C88 strain was also evaluated considering the cell surface properties. The enzymatic and chemical treatments of bacterial cells caused a decrease in the ability of the LAB strain to capture free radicals. It was shown that there is a relationship between protein components on the cell surface and the antioxidant activity of bacteria [23,53].

### ABTS radical scavenging activity

**Fig 3:**
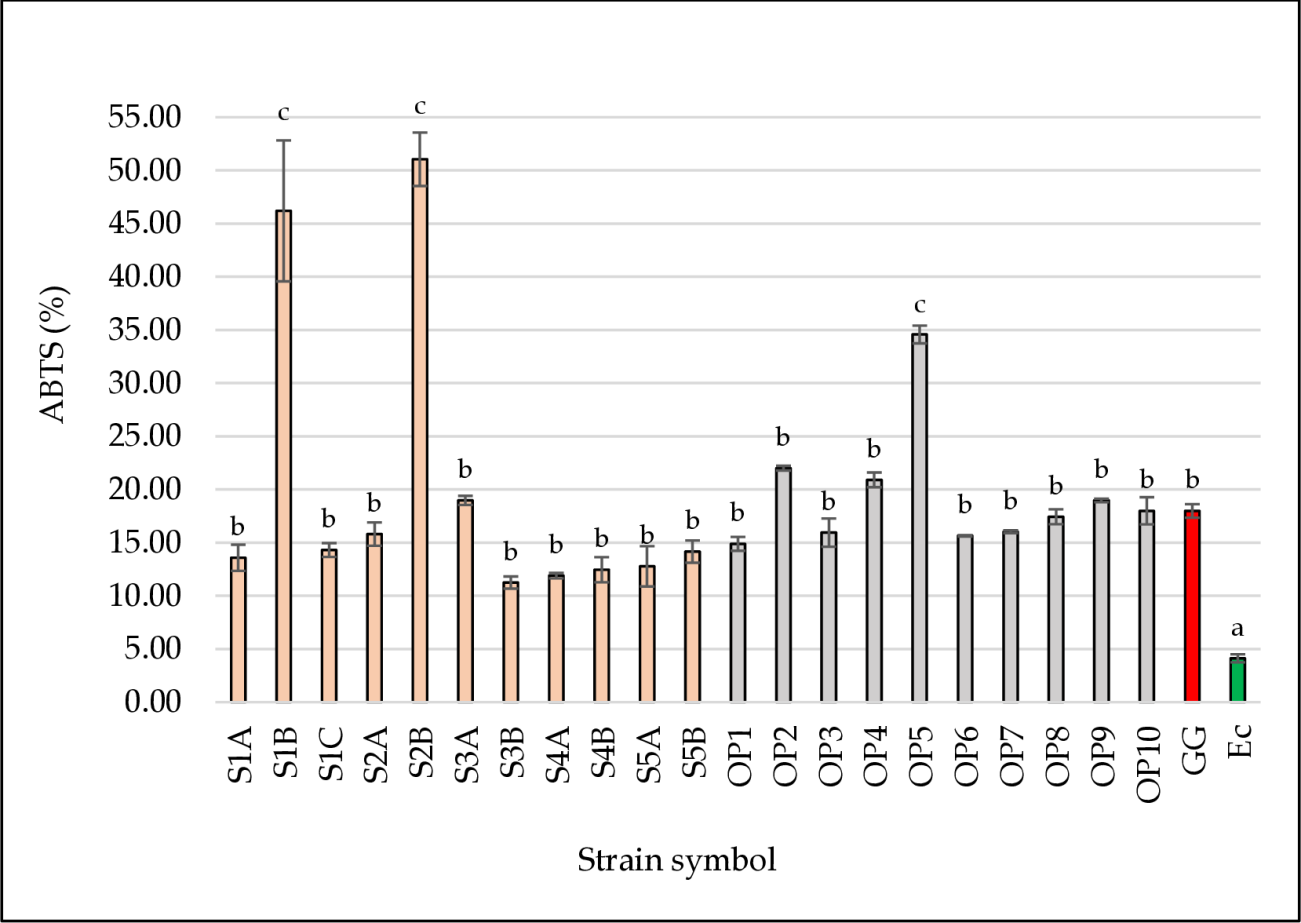
Antioxidant activity by the ABTS method of the tested LAB strains. The figure presents the ability of bacterial strains to capture free ABTS radicals. Strains isolated from beef hams are marked in pink. Strains isolated from the meat factory environment are marked in grey. The reference probiotic strain *L. rhamnosus* GG (positive control) is marked in red. The reference strain *E. coli* DH5α (negative control) is marked in green. ^a,b,c^ Means in the same column followed by different superscripts are significantly different (*p* < 0.05).

The ABTS assay is a spectrophotometric method in which antioxidants react with the oxidized ABTS cation radical, which causes its reduction and, consequently, a change in the color of the solution [54]. Total antioxidant activity determined by the ABTS^+^ method showed different results (Fig 3). The highest, statistically significant antioxidant activity was shown by strains *L. plantaru*m S1B, *L. pentosus* S2B, and *L. plantarum* OP5 (34.57–51.05%; *p* < 0.05). This activity was also significantly (*p* < 0.05) higher than the activity of the reference strain *L. rhamnosus* GG (17.98%). In the study of Shi et al. [35], a different, higher ABTS radical scavenging ability of *L. rhamnosus* GG strain was obtained compared to our study (34.00%, 17.98%, respectively). The tested LAB strains had different abilities (0.00–55.38%), but eight strains were distinguished representing higher results than the reference strain. Fifteen LAB strains were tested for free radical scavenging using the ABTS method [55]. The capacity was 12.1–47.1% and *L. reuteri* MG5149 showed the highest antioxidant activity. In the study by Abduxukur et al. [21] strains of the genera *Lactobacillus* and *Enterococcus* were tested in the ABTS^+^ assay. The results ranged from 20 to over 40% free radical scavenging activity. Interestingly, researchers tested the antioxidant properties of intact cell suspensions and cell-free extracts of microorganisms. The studies showed that cell-free extracts were characterized by greater antioxidant activity. The same conclusions were drawn by Song et al. [56], wherein the ABTS^+^ assessment, *L. rhamnosus* GG and *L. brevis* KCCM 12203P also showed greater antioxidant activity for heat-killed bacteria.

Considering our results from Fig 2 and Fig 3, it can be stated that the antioxidant activity between the tests differs. Floegel et al. [57] explain this phenomenon by the specificity of the tests. The ABTS^+^ test applies to both hydrophilic and lipophilic systems, while the DPPH test works better in hydrophobic systems. According to Zhou et al. [58], free radical scavenging methods can be successfully used to evaluate the antioxidant activity of lactic acid bacteria. The most commonly used methods for testing are intact cells, cell-free extracts, cell lysates, and post-fermentation metabolites. The obtained results suggest that lactic acid bacteria strains whose cell-free supernatant is rich in bioactive compounds can effectively neutralize free radicals and improve antioxidant properties. Wu et al. [49] observed a relationship between antioxidant activity and a fraction of the same strains. Antioxidant activity was mainly concentrated in Cell-Free Fermentation Supernatants (CFS), with only a few LAB strains showing minimal reducing activity in Intact Cells (IC), and no activity was detected in Cell-Free Extracts (CFE). According to the authors, key antioxidant enzymes as well as non-enzymatic metabolites (for example exopolysaccharides, bioactive peptides, or short-chain fatty acids) are found in the CFS fraction, which play a key role in free radical scavenging [49].

### Superoxide anion scavenging test

**Fig 4:**
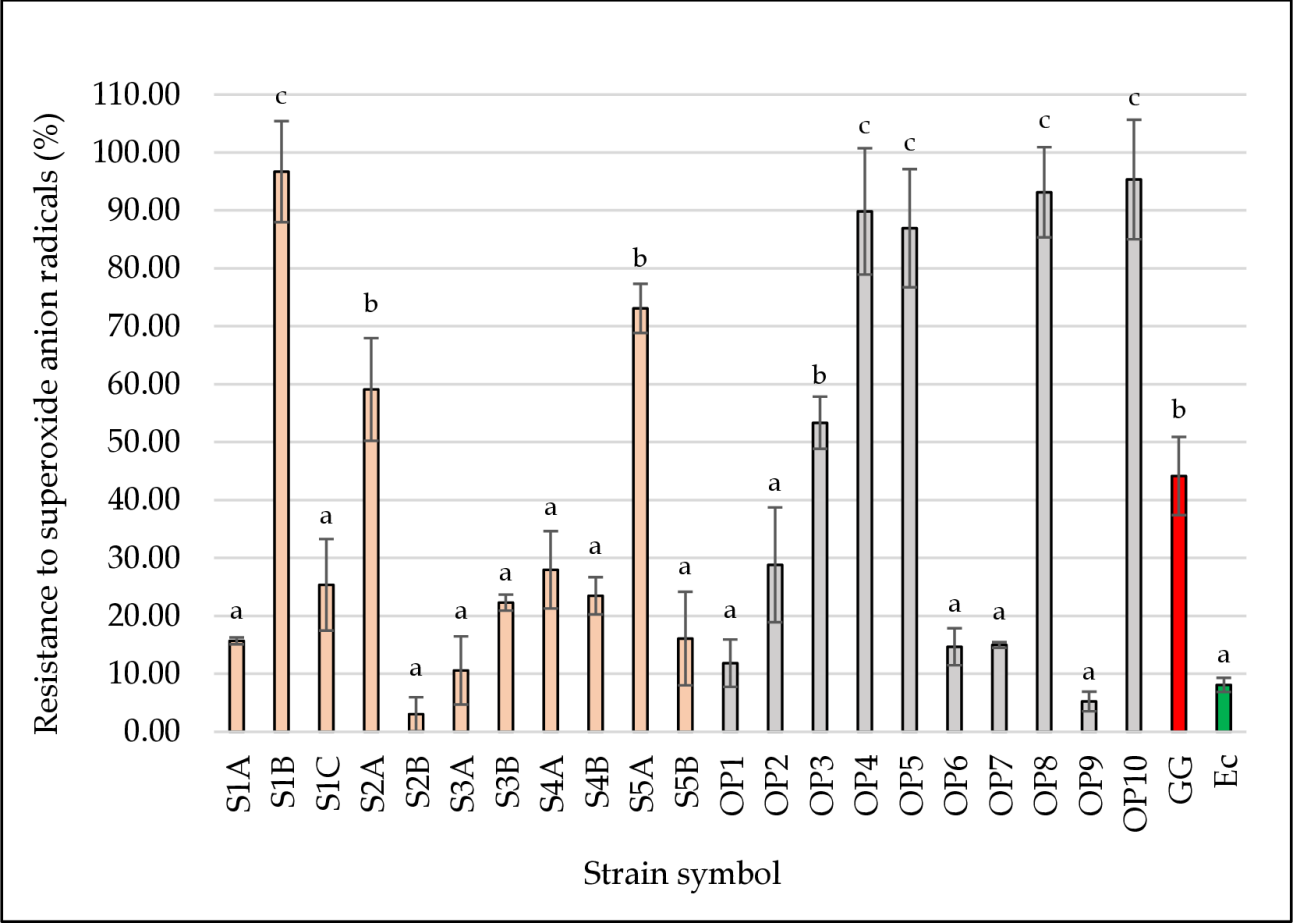
Resistance to superoxide anion radicals of the tested LAB strains. The figure presents the ability of bacterial strains to capture free superoxide anion radicals. Strains isolated from beef hams are marked in pink. Strains isolated from the meat factory environment are marked in grey. The reference probiotic strain *L. rhamnosus* GG (positive control) is marked in red. The reference strain *E. coli* DH5α (negative control) is marked in green. ^a,b^ Means in the same column followed by different superscripts are significantly different (*p* < 0.05).

Superoxide anion (O_2_^•−^) is a reduced form of molecular oxygen O_2_ and is one of the most important reactive oxygen species. It is responsible for oxidative stress in organisms and is produced as a byproduct of the mitochondrial respiratory chain [59]. The superoxide anion radical scavenging activity may be closely related to the production of the superoxide dismutase enzyme [60]. Fig 4 presents the resistance to superoxide anion radicals of the tested LAB strains. Strains *L. plantarum* S1B, OP4, OP5, OP8, and OP10 were found to exhibit significantly (*p* < 0.05) higher resistance to superoxide anion radicals (86.93–96.70%) than *L. rhamnosus* GG (44.15%). The results are consistent with the findings of our previous report [26], in which strains isolated from fermented loins, gammons, and sausages, showed resistance to superoxide anion radicals ranged from 20.34 to 96.52%, with the highest value for *L. plantarum* SCH1, *Pd. pentosaceus* BAL6 and KL14 (62.08–96.52%). A similar value was also performed for *L. rhamnosus* GG (43.45%). In the study by Shi et al. [35] the ability of LAB strains to capture superoxide anion was presented and the values were 59.27–86.84%. *L. rhamnosus* was assessed at 76.56%, which is much higher than that reported in our study.

### Hydroxyl radical resistance

**Fig 5:**
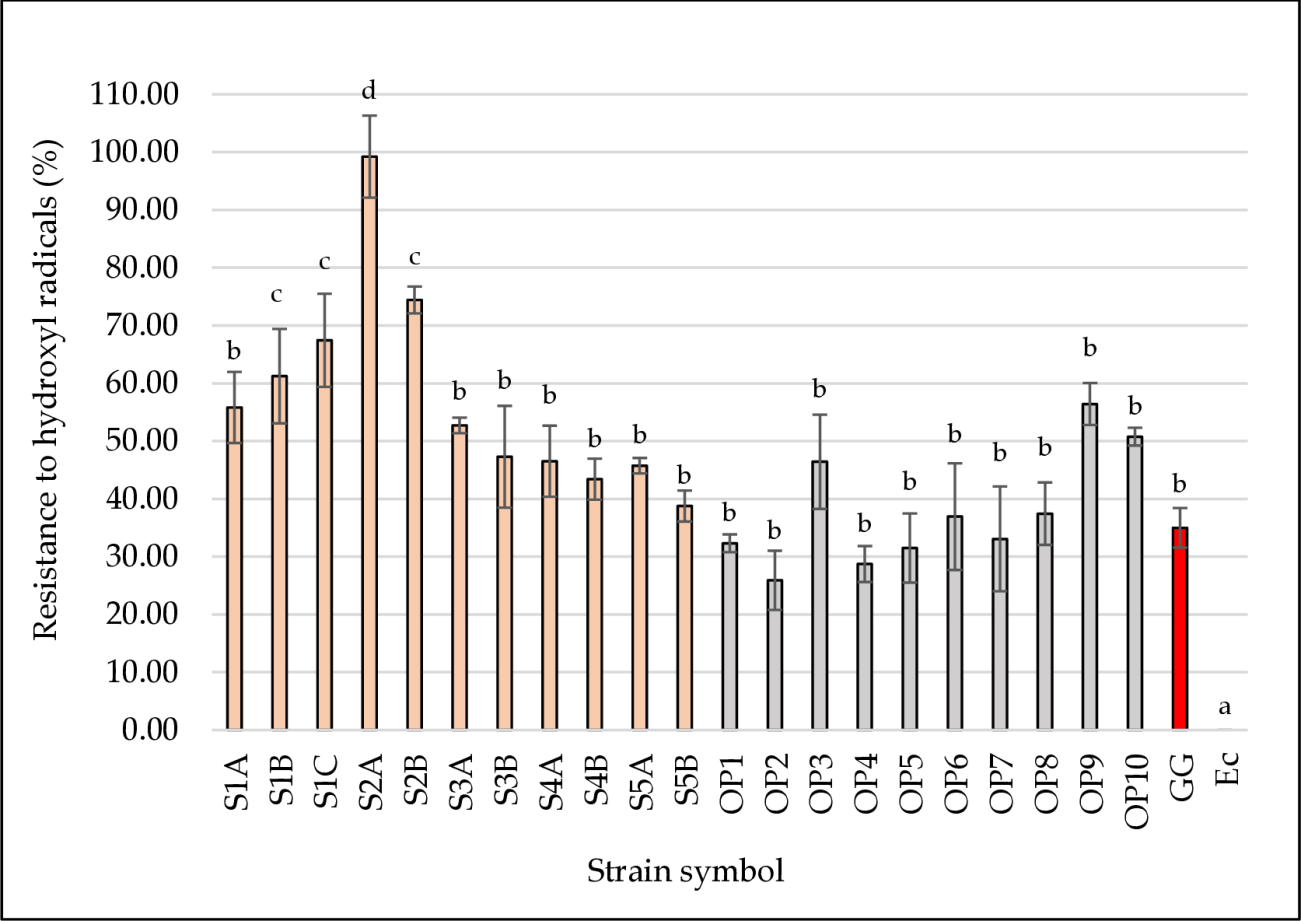
Resistance to hydroxyl radicals of the tested LAB strains. The figure presents the ability of bacterial strains to capture hydroxyl radicals. Strains isolated from beef hams are marked in pink. Strains isolated from the meat factory environment are marked in grey. The reference probiotic strain *L. rhamnosus* GG (positive control) is marked in red. The reference strain *E. coli* DH5α (negative control) is marked in green. ^a,b,c,d^ Means in the same column followed by different superscripts are significantly different (*p* < 0.05).

The hydroxyl radical is one of the most reactive and one of the most harmful radicals found in biological systems. It causes damage to DNA, proteins, and membranes [5]. They mainly originate from the Fenton reaction in the presence of transition metals (Fe^2+^ and Cu^2+^). The ability of some antioxidants to chelate these ions may lead to the inhibition of the formation of hydroxyl radicals [23]. The resistance to hydroxyl radicals of the tested LAB strains is presented in Fig 5. Significantly the highest resistance to hydroxyl radicals was characterized by four strains: *L. plantarum* S1B, S1C, S2A, and *L. pentosus* S2B (61.24–99.22%, *p* < 0.05). The reference strain *L. rhamnosus* GG showed a resistance of 34.99%. Düz et al. [61] suggest that the strong hydroxyl radical scavenging activity of LAB strains, including *L. plantarum*, may be related to the ability to bind metal ions, such as Fe^2+^. Among the tested strains, the highest value of resistance to hydroxyl radicals was represented by strains of the *L. plantarum* species. The report of Xu et al. [62] stated that extracellular polysaccharides (EPS) derived from the supernatant showed strong antioxidant activity and could scavenge hydroxyl radicals. Since the antioxidant activity of the supernatant was also examined in our study, it can be assumed that our isolates could produce EPS. In the study by Hu et al. [63], fifteen LAB strains were isolated from “Jiangshui,” pickles, and feces. Similar to our study, hydroxyl-free radical scavenging capacity was compared with *L. rhamnosus* GG. The values ranged from 21.07 to 62.80%, with a value of 24.67% for *L. rhamnosus* GG. In our study, hydroxyl-free radical scavenging capacity was 25.90–99.22%, with most strains exhibiting higher values than the reference strain (34.99%).

### Superoxide dismutase (SOD) and catalase (CAT) activity

LAB have antioxidant activity in the human intestine also due to the production of antioxidant enzymes. Antioxidant enzymes are activated under oxidative stress conditions, which allows lactic acid bacteria to resist oxidative stimulation [64]. The most important antioxidant enzymes include superoxide dismutase (SOD). It is a metalloenzyme with subunit structural organization that is the main regulator of oxidative processes in biological cells [48]. SOD catalyzes the dismutation reaction of the superoxide anion radical to less reactive hydrogen peroxide and oxygen. Thus, it reduces the intracellular concentration of free metal cations and alleviates the damage caused by hydrogen peroxide [1]. Table 3 presents the results of superoxide dismutase activity. The highest SOD activity in the colorimetric tests was shown by *L. pentosus* S2B, *L. plantarum* OP5, and OP8 (3.56–3.78 U/mL; *p* < 0.05). *L. rhamnosus* GG had low SOD activity of 0.45 U/mL. In the semiquantitative Peroxide Test at time 0, all LAB strains and the *E. coli* strain were unable to produce H_2_O_2_ (0 mg/L H_2_O_2_). After 24 h of the experiment, a small amount of H_2_O_2_ was produced by *L. pentosus* S2B, *L. plantarum* S3A, and S4A strains, amounting to 1 mg/L H_2_O_2_. In the studies by Tomusiak-Plebanek et al. [37], 25 *Lactobacillus* strains were subjected to antioxidant tests. In SOD kits, superoxide dismutase activity was observed in *Lactobacillus (L.) delbrueckii* subsp. *delbrueckii*, *Lactobacillus (L.) acidophilus*, and *Limosilactobacillus (L.) fermentum* (0.22–1.40 U/mL), but most of the tested LAB strains did not show any SOD activity. Significantly higher activity was observed in several LAB strains from our studies. Interestingly, according to Aziz et al. [4], one of the forms of superoxide dismutase Cu/Zn-SOD is found in quite high concentrations in *E. coli* proteins. This explains the SOD activity in our study, which was found in *E. coli* DH5α (0.42 U/mL). In the semiquantitative Peroxide Test Strip method, selected strains of *L. delbrueckii* and *L. acidophilus* showed SOD activity after 2 and 24 h of incubation. This activity was not observed in strains of the *L. plantarum* species. In our study, SOD activity in *L. plantarum* strains was negligible (1 mg/L) or not detected at all. There are four types of SOD, which differ in the metal atoms present in their active center: iron-containing (Fe-SOD), manganese-containing (Mn-SOD), copper/zinc-containing (Cu/Zn-SOD), and nickel-containing (Ni-SOD). Most strictly anaerobic microorganisms possess Fe-SOD. In the genera, *Streptococcus* and *Lactococcus*, Mn-SOD activity is found. In contrast, some *E. coli* bacteria even have several types. Most lactobacilli do not possess any SOD activity [20], which was also observed in our studies. Lactic acid bacteria are microorganisms that do not require oxygen for growth. LAB can produce protection against the harmful effects of oxygen, which is toxic to them. The species *L. casei* and *L. paracasei* accumulate manganese, which allows for the effective elimination of superoxide anions during aerobic growth. This is possible thanks to the system of numerous manganese transport proteins of the NRAMP (Natural Resistance-Associated Macrophage Proteins) and ABC (ATP-Binding Cassette) types. In turn, the ability to accumulate manganese is associated with the lack of true SOD activity [20,65]. Our strains *L. pentosus* S2B, *L. plantarum* OP5, and OP8 belong to the same genus *Lactiplantibacillus*. In the study by Gao et al. [66], the complete genome of *L. plantarum* Y44 was investigated. Y44 was shown to have SOD activity, but no genes encoding SOD were found. Its lack is compensated by high intracellular manganese (Mn^2+^) accumulation. The manganese accumulation system may enhance the tolerance of *L. plantarum* to oxidative stress [67]. Perhaps a similar phenomenon can be observed in our strains. However, it is necessary to sequence the whole genome, which we plan to do in the future.

**Table 3:** Superoxide dismutase (SOD) activity and catalase activity (CAT) of the tested LAB strains.

Strain symbol	Superoxide dismutase (SOD) activity			Catalase (CAT) activity	
	DetectX^®^ SOD kits (U/mL)	MQuant^®^ (mg/L H_2_O_2_)		MQuant^®^ (mg/L H_2_O_2_)	
		0 h	24 h	0 h	24 h
S1A	0.70 ± 0.10^a^	0	0	30	30
S1B	0.34 ± 0.14^a^	0	0	30	30
S1C	0.00 ± 0.00^a^	0	0	30	30
S2A	1.23 ± 0.13^b^	0	0	30	30
S2B	3.70 ± 0.06^c^	0	1	30	30
S3A	2.78 ± 0.20^b^	0	1	30	30
S3B	0.54 ± 0.08^a^	0	0	30	30
S4A	2.78 ± 0.26^b^	0	1	30	30
S4B	0.10 ± .0.02^a^	0	0	30	30
S5A	0.68 ± 0.12^a^	0	0	30	30
S5B	2.10 ± 0.30^b^	0	0	30	30
OP1	0.15 ± 0.05^a^	0	0	30	30
OP2	0.98 ± 0.24^a^	0	0	30	30
OP3	1.88 ± .0.24^b^	0	0	30	30
OP4	0.13 ± 0.01^a^	0	0	30	30
OP5	3.78 ± 0.02^c^	0	0	30	30
OP6	0.05 ± 0.00^a^	0	0	30	30
OP7	0.88 ± 0.06^a^	0	0	30	30
OP8	3.56 ± 0.14^c^	0	0	30	30
OP9	0.03 ± .0.00^a^	0	0	30	30
OP10	0.06 ± 0.02^a^	0	0	30	30
GG	0.45 ± 0.15^a^	0	0	30	30
Ec	0.42 ± 0.02^a^	0	0	30	10

a,b,c Means in the same column followed by different lowercase letters between the strains are significantly different (*p* < 0.05).

Catalase (CAT) is an enzyme from the oxidoreductases that catalyze the decomposition of hydrogen peroxide into water and oxygen. Catalases and peroxidases are practically absent in LAB [1]. In this study, a test for catalase activity was also performed in phenotypic studies (Table 1). The tested strains, like most lactic acid bacteria, are catalase-negative. In experiments with Peroxide test MQuant^®^, all LAB strains did not reduce the amount of hydrogen peroxide added to MRS broth (Table 3). Only the *E. coli* strain reduced the amount of hydrogen peroxide added to LB broth from 30 mg/L to 10 mg/L after 24 h of incubation. Most *E. coli* strains are catalase-positive [68]. There are two types of catalases: true heme-dependent catalase and manganese-containing pseudocatalases. True heme-dependent catalases have been detected in many LAB species, but only with the addition of heme or hematin in the substrate. Pseudocatalases (non-heme catalases) do not require heme or hematin for their activity but are in turn rarely found in LAB [20]. Some non-heme catalases (MnKat) have been discovered in *L. plantarum* [69], but we cannot detect such activity in our strains.

#### Hydrogen peroxide resistance.

In the screening studies, the use of hydrogen peroxide as an oxidant is particularly useful [64]. So, the next experiment concerned the evaluation of the resistance of the strains to hydrogen peroxide present at three different concentrations: 0.4 mM H_2_O_2_, 0.7 mM H_2_O_2_, and 1.0 mM H_2_O_2_ (Table 4). Initial optical density OD_600_ did not differ statistically with any significance between strains (OD_600_ 2.203–2.797; *p* > 0.05). The concentration of 0.4 mM H_2_O_2_ significantly reduced OD_600_ for most strains, except *L. plantarum* OP5 and *E. coli* (*p* < 0.05). The application of higher concentrations of H_2_O_2_ (0.7 mM and 1.0 mM) caused a further decrease in the optical density of strains. Strains *L. plantarum* S1A, *L. argentoratensis* S5B, *L. plantarum* OP4, and OP8 showed statistically significant greatest resistance to 1.0 mM hydrogen peroxide (OD_600_ 1.324–1.398; *p* < 0.05) and at the same time showed better resistance than *L. rhamnosus* GG (OD_600_ 1.301). With increasing hydrogen peroxide concentration, the duration of the lag phase is prolonged, which indicates that the presence of hydrogen peroxide causes oxidative damage, which consequently leads to inhibition of bacterial growth [70]. Numerous studies have been documented on H_2_O_2_-resistant lactic acid bacteria strains. Hu et al. [63] demonstrated moderate resistance of lactobacilli isolates to H_2_O_2_ in the presence of 1, 2, and 3 mmol/L hydrogen peroxide. Similar results were reported by Li et al. [23], where *L. plantarum* strains lost viability upon exposure to various concentrations of hydrogen peroxide. In the study by Tang et al. [70], the results showed that *L. plantarum* MA2 was highly tolerant to very high concentrations of hydrogen peroxide (2.0 mM).

**Table 4:** Hydrogen peroxide resistance of the tested strains.

Strain symbol	Hydrogen peroxide resistance (OD_600_)			
0.0 mM H_2_O_2_	0.4 mM H_2_O_2_	0.7 mM H_2_O_2_	1.0 mM H_2_O_2_
S1A	2.768 ± 0.021^aB^	1.424 ± 0.009^aA^	1.370 ± 0.008^cA^	1.328 ± 0.058^dA^
S1B	2.797 ± 0.182^aC^	1.459 ± 0.050^aB^	0.724 ± 0.022^bA^	0.450 ± 0.052^bA^
S1C	2.696 ± 0.041^aC^	1.318 ± 0.021^aB^	1.319 ± 0.045^cB^	0.868 ± 0.004^cA^
S2A	2.746 ± 0.174^aC^	1.198 ± 0.018^aB^	1.151 ± 0.020^cB^	0.658 ± 0.033^cA^
S2B	2.736 ± 0.190^aC^	1.112 ± 0.015^aB^	1.103 ± 0.009^cB^	0.380 ± 0.037^bA^
S3A	2.710 ± 0.251^aC^	1.146 ± 0.052^aB^	0.954 ± 0.023^bB^	0.367 ± 0.016^bA^
S3B	2.749 ± 0.188^aC^	1.180 ± 0.042^aB^	0.930 ± 0.039^bB^	0.353 ± 0.037^bA^
S4A	2.740 ± 0.177^aC^	1.293 ± 0.037^aB^	1.073 ± 0.022^cB^	0.798 ± 0.088^cA^
S4B	2.623 ± 0.346^aC^	1.474 ± 0.058^aB^	1.144 ± 0.015^cA^	1.011 ± 0.009^dA^
S5A	2.342 ± 0.017^aC^	1.162 ± 0.016^aB^	1.122 ± 0.004^cB^	0.405 ± 0.006^bA^
S5B	2.651 ± 0.236^aB^	1.617 ± 0.081^bA^	1.487 ± 0.034^cA^	1.344 ± 0.036^dA^
OP1	2.470 ± 0.061^aC^	1.907 ± 0.072^bB^	1.564 ± 0.029^dB^	1.295 ± 0.007^dA^
OP2	2.203 ± 0.062^aC^	1.785 ± 0.034^bB^	1.742 ± 0.010^dB^	1.120 ± 0.011^dA^
OP3	2.475 ± 0.131^aC^	1.940 ± 0.045^bB^	1.116 ± 0.012^cA^	1.029 ± 0.014^dA^
OP4	2.306 ± 0.066^aC^	1.964 ± 0.031^bB^	1.894 ± 0.004^dB^	1.398 ± 0.007^dA^
OP5	2.452 ± 0.022^aB^	2.147 ± 0.018^cB^	1.308 ± 0.047^cA^	1.195 ± 0.011^dA^
OP6	2.575 ± 0.038^aC^	2.170 ± 0.011^cB^	1.314 ± 0.014^cA^	1.271 ± 0.006^dA^
OP7	2.552 ± 0.045^aC^	2.140 ± 0.009^cB^	1.328 ± 0.006^cA^	1.290 ± 0.067^dA^
OP8	2.548 ± 0.043^aC^	2.146 ± 0.010^cB^	1.337 ± 0.010^cA^	1.324 ± 0.018^dA^
OP9	2.533 ± 0.034^aC^	2.158 ± 0.011^cB^	1.280 ± 0.007^cA^	1.260 ± 0.008^dA^
OP10	2.517 ± 0.033^aC^	2.101 ± 0.002^cB^	1.199 ± 0.012^cA^	1.173 ± 0.012^dA^
GG	2.535 ± 0.045^aC^	1.965 ± 0.015^bB^	1.443 ± 0.033^cA^	1.301 ± 0.002^dA^
Ec	2.750 ± 0.020^aB^	2.020 ± 0.025^cB^	0.238 ± 0.002^aA^	0.110 ± 0.004^aA^

^a,b,c,d^ Means in the same column followed by different lowercase letters between the strains are significantly different (*p* < 0.05).

^A,B,C,D^ Means in the same row followed by different uppercase letters between the treatments are significantly different (*p* < 0.05).

All of the tested LAB strains are catalase-negative, which means that they cannot degrade hydrogen peroxide directly. As presented in Table 4, hydrogen peroxide effectively inhibits the number of viable LAB cells. However, although lactobacilli are catalase-negative by nature, they develop a kind of resistance to hydrogen peroxide, which may be the result of gene expression [51,71]. The presence of catalase genes and H_2_O_2_ degradation may therefore contribute to the protection of bacteria and/or the evolution towards an aerobic lifestyle of anaerobic and microaerophilic microorganisms. Hydrogen peroxide is a rather weak oxidant, but it can cause the formation of hydroxyl radicals, which, as mentioned earlier, are strong oxidants. According to Li et al. [23], many *Lactobacillus* strains are resistant to hydrogen peroxide to varying degrees. Considering the results in Table 4, it can be concluded that the strains isolated from the production environment are more resistant to high concentrations of hydrogen peroxide, while the strains isolated from fermented hams are more sensitive to it.

### Cluster analysis

**Fig 6:**
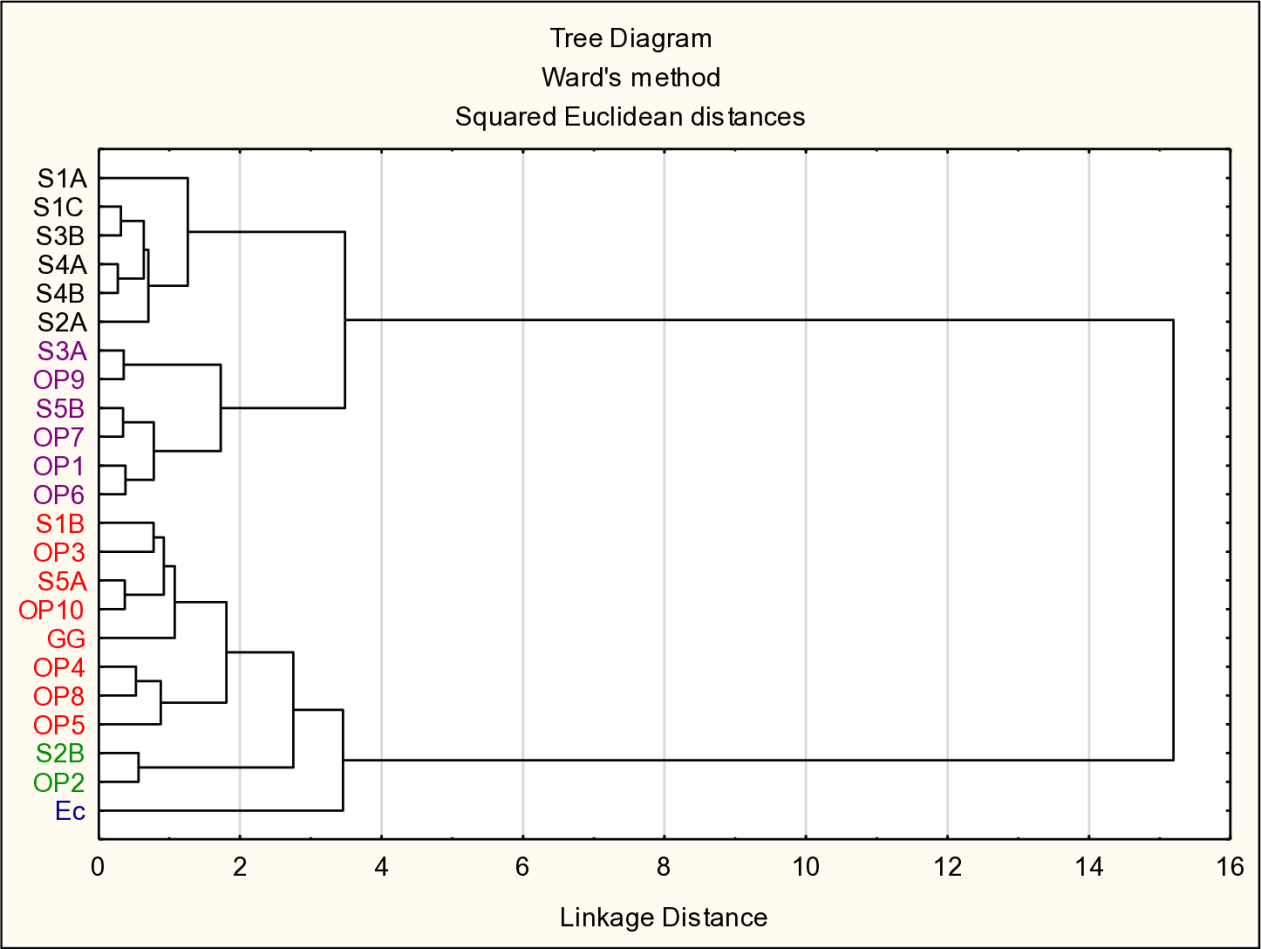
Cluster analysis using Ward’s method. The figure shows five isolated clusters. Cluster 1 is marked in blue. Cluster 2 is marked in green. Cluster 3 is marked in black. Cluster 4 is marked in purple. Cluster 5 is marked in red. The assignment of strains to clusters is not conditioned by the source of isolation, genus, or species of bacteria.

Fig 6 presents a Cluster analysis using Ward’s method. The analysis identified five clusters. Cluster 1 represented the reference strain of *Escherichia coli* (Ec), which was a negative control in our study. Cluster 2 distinguished two strains *L. pentosus* S2B and *Pd. pentosaceus* OP2. Cluster 3 consisted of six strains isolated from the pork hams (*L. plantarum* S1A, S1C, S2A, S4A, and *L. pentosus* S3B, S4B). Cluster 4 consisted of strains *L. plantarum* S3A, OP1, OP6, OP9, and *L. argentoratensis* S5B, OP7. Cluster 5 represented strains most similar to the reference strain *L. rhamnosus* GG (positive control) and were *L. plantarum* S1B, S5A, OP4, OP5, OP8, OP10, and *L. paracasei* OP3. The strains were not grouped by the source of isolation, nor by genus or species. This indicates that antioxidant properties are strain-dependent.

## Conclusions

To maintain optimal functioning of the body, the consumption of natural antioxidants is becoming an increasingly popular option, which allows for improved mechanisms of protection against free radicals. Microorganisms and their metabolites, which can be consumed in food or as dietary supplements, have become an important and interesting topic. Consuming foods, especially fermented foods, containing antioxidants can reduce the level of reactive oxygen species in tissues and significantly reduce the risk of degenerative diseases caused by oxidative stress. Lactic acid bacteria are excellent carriers of antioxidants. This is especially true for antioxidant defense achieved through the production and regulation of antioxidant enzymes. Active post-fermentation metabolites have a beneficial effect on human health by modulating the gut microbiota, scavenging free radicals, and chelating metal ions.

The antioxidant activity of lactic acid bacteria strains varies depending on the test used. Therefore, to screen the bacteria, we used different methods to compare their antioxidant properties and to be able to assess in detail the effects of scavenging reactive free oxygen radicals, as well as the ability to synthesize antioxidant enzymes. A total of twenty-one lactic acid bacteria isolates were tested, which were compared with two reference strains. The tested strains demonstrated high tolerance to different H_2_O_2_ levels and strong scavenging capacities to various free radicals, including DPPH, ABTS, superoxide anions, and hydroxyl radicals. The activity of superoxide dismutase was confirmed. Such strains of lactic acid bacteria with desirable antioxidant properties may be a promising material for both microbiology and the food industry. Lactic acid bacteria with antioxidant properties may play an important role in the prevention and treatment of diseases associated with oxidative stress. Their ability to neutralize free radicals, reduce reactive oxygen species, and protect cellular structures from oxidative damage makes them a promising tool in supporting gut health and the overall metabolic balance of the body.

Further studies are undoubtedly necessary to clarify the mechanisms involved in antioxidant processes. The present studies only confirm the efficacy *in vitro*, and therefore *in vivo* studies are necessary to fully assess the ability of lactic acid bacteria strains to inhibit oxidative stress, especially because the human microbiota must be resistant to it. In the future, it is necessary to perform tests on cell lines, as well as genetic tests confirming the presence of appropriate genes in the bacterial genome. Selected strains will be analyzed by whole genome sequencing. Careful consideration of the most technically suitable lactic acid bacteria strains may prove to be the key to success when antioxidant intervention is required.

## Supporting information

S1 Table Antioxidant activity. (XLSX)
